# A Rare Malignancy of the Eyelid: Report A Case of Primary Periocular Histiocytoid Carcinoma

**DOI:** 10.30699/ijp.2024.2016655.3219

**Published:** 2024-07-24

**Authors:** Arezu Rahnavard, Elham Mirzaian, Reyhaneh Safaei, Ida Mehrabi

**Affiliations:** 1 Department of Pathology, Tehran University of Medical Sciences, Tehran, Iran; 2 Department of Pathology, Shariati Hospital, School of Medicine, Tehran University of Medical Sciences, Tehran, Iran; 3 Department of Pathology, Faculty of Veterinary Medicine, University of Tehran, Tehran, Iran; 4 Department of Pathology, Tehran University of Medical Sciences, Tehran, Iran

**Keywords:** Eyelid malignancy, Histiocytoid carcinoma, Orbital malignancy, Periorbital malignancy, PPHC

## Abstract

Primary periocular histiocytoid carcinoma is a very rare malignant tumor. Until now, less than 50 cases have been reported in the English literature. It is characterized by resistant epiphora, limitation in extraocular motility, and ptosis. The definitive diagnosis of this lesion is made based on detecting histological histiocytoid features along with tracing positivity of specific biomarkers using immunohistochemistry. However, pathologists may be faced with two major obstacles in the diagnosis of this tumor including distinguishing it from metastatic histiocytoid lesions and also from benign mimics such as reactive inflammatory lesions. Here, we describe a case of primary periocular histiocytoid carcinoma located on the eyelid as well as review the literature to clarify the histopathological and diagnostic features of this tumor.

## Introduction

Primary periocular histiocytoid carcinoma (PPHC) is a very rare malignant tumor that often arises from orbit and/or eyelid as a primary lesion, but it may be metastatic from other organs such as the breast (1). This carcinoma is more common in older men (2). PPHC has an aggressive and metastatic behavior, it can invade the surrounding lymph nodes and have a poor prognosis; even leading to the patient’s death (3). It is characterized by a painless periorbital diffuse swelling, thickening, and infiltration around the eyelid (4). Clinically, this tumor is commonly mistaken for benign and inflammatory lesions including chronic blepharoconjunctivitis or chalazion, thus causing a delayed diagnosis and management of patients (5). Unfortunately, distinguishing between non-neoplastic, primary neoplastic, and metastatic histiocytoid lesions (especially of breast origin) only based on immunohistochemistry and molecular studies is very difficult for both pathologists and clinicians. Such differentiation is of tremendous importance due to the huge difference in the course, treatment options, and management approaches of these diseases. Herein, we describe a case of PPHC located on the eyelid with a review of similar previously reported cases describing their different aspects.

## Case Presentation

**Fig. 1:**
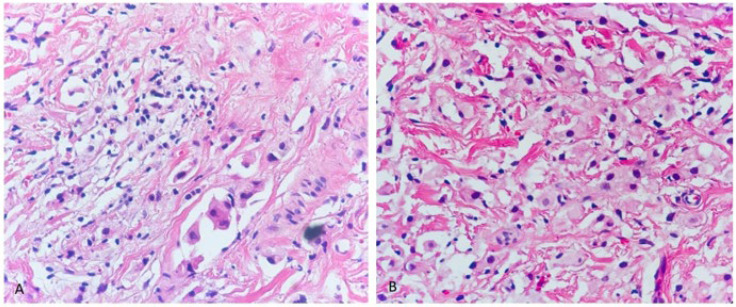
A, B. H&E-stained slide showing infiltrating isolated and small aggregates of cells with mild nuclear atypia and foamy vacuolated cytoplasm (original magnification *40)

**Fig. 2:**
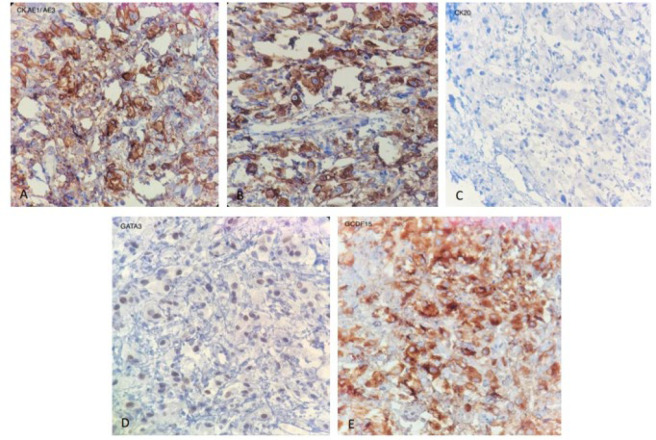
Immunohistochemical studies reveal: A. Positive staining for AE1/AE3. B. Positive staining for CK7. C. Negative staining for CK20. D. Positive staining for GATA3 and E. Positive staining for GCDEP15

The case described was a 58-year-old man who presented with resistant epiphora and swelling in the right lower eyelid three years and 6 months before seeking medical attention, respectively. The patient's past medical history was insignificant. Due to the progress and persistence of the mentioned clinical manifestations, the patient was referred to our clinic. Examination revealed partial extra-ocular movement limitation, as well as ptosis. Increased soft tissue density in the right orbit and proptosis in the paranasal site were also detected on a computed tomography (CT) scan. In an evaluation by positron emission tomography (PET) scan, no abnormality was found except for the mentioned lesion in the right eyelid. An incisional biopsy was performed, and the specimen was submitted for pathologic evaluation. Microscopic examination showed a fibrous connective tissue infiltrated by isolated and small aggregates of cells with mild nuclear atypia and foamy vacuolated cytoplasm indicating histiocytoid features (Figure 1). In the immunohistochemical studies, the tumor cells were positive for CK AE1/AE3, CK7, GCDFP15, and GATA3, while CK20, TTF1, CDX2, and Mammaglobin were negative (Figure 2). At last, based on Immunohistomorphological findings and PET scan results showing no evidence of tumoral lesions in any other site, a diagnosis of primary periocular histiocytoid carcinoma was made for this patient.

## Discussion

Primary periocular histiocytoid carcinoma is a rare aggressive tumor that usually originates from the periocular region. Prominent morphological features include vacuolated cytoplasm along with nuclear atypia (6). Unfortunately, differentiation between the primary and the metastatic lesions (especially of breast origin) through microscopic and immunohistochemical examinations is very challenging (7,8). The differential diagnosis should also take into consideration the possibility of primary or metastatic cutaneous adnexal carcinoma, including cutaneous apocrine carcinoma with signet ring/histiocytoid features and histiocytoid melanoma. To distinguish Primary periocular histiocytoid carcinoma from cutaneous adnexal lesions, relying solely on morphological and immunohistochemical evaluation may not yield conclusive results. Therefore, it is imperative to consider the tumor location and establish a clinicopathologic correlation for accurate diagnosis. (9). Microscopically, both primary and metastatic tumors are characterized by proliferation of round to oval cells with histiocytoid cytoplasm showing signet-ring or vesicular features (10) as well as positivity for various apocrine or epithelial markers such as AE1/AE3, GCDFP 15, CK7, Ber-EP4, EMA, and CAM 5.2 and negativity for CK20, TTF1, and CDX2 (11). A positive staining for pan-cytokeratin and a lack of melanocytic markers aid in differentiating these conditions from melanoma (9). However, some biomarkers that are positive specifically in primary tumors may be helpful in the differentiation of these two entities. The markers include E-cadherin which is positive in 80% of the primary lesions while only positive in 25% of metastatic types (12) The other diagnostic challenge is the similarity of the tumoral and inflammatory cells such as macrophages, which may misdirect pathologists to make a diagnosis of reactive inflammatory diseases. From a histogenesis point of view, while the exact source is still unclear, some evidence suggests eccrine sweat glands as the origin of the tumoral cells given the presence of intracytoplasmic lumina with villi-like features in ultrastructural assessments (13). Others propose an apocrine origin due to the existence of smooth and rough endoplasmic reticulum, lipid droplets, and positive GCDFP-15 marker that is negative in eccrine gland cells (14). As we previously mentioned it is challenging to differentiate between the primary and metastatic types only based on histology or IHC study, however, the following criteria may be beneficial: 

1) Metastatic types are usually associated with a positive family history of breast cancer.

2) Metastatic types are usually bilateral, while the primary type is often unilateral. 

3) Unlike the primary periocular histiocytoid carcinoma, in metastatic type evidence of metastasis of the primary tumor to the other organs rather than orbit might be present as well; for instance, in the case of breast cancer, involvement of the liver, bones or lungs can be a clue. 

Only a few cases of primary periocular histiocytoid carcinoma have been previously described. In a similar case reported by Yates* et al.* in 2019 (15), the patient was a male suffering from a gradual reduction in visual acuity with a sensation of a mass in the right eye. Limitations of extraocular motility and ptosis were identified in physical examination. Magnetic resonance imaging revealed a small enhancing mass in the orbit with expansion to the globe. Microscopic examination showed histological features correlated with histiocytoid carcinoma that were ultimately confirmed by immunohistochemistry. In another case described by Palakkamanil* et al.* in 2020 (11), a 73-year-old man presented with an upper lateral orbital rim mass. The pathologic evaluation revealed diffuse and deeply infiltrating tumor cells extending through the dermis to subcutis, orbicular muscle bundles, and nerve fibers; the tumor cells had monotonous histiocytoid appearance with foamy granular eosinophilic cytoplasm. In the IHC study, a positivity for AE1/AE3, CK7, GCDFP-15, E-cadherin, androgen receptor, and GATA3 was reported. 

## Conclusion

The rareness of this tumor often causes delays in the diagnosis and management of patients. The purpose of this case report is to familiarize both clinicians and pathologists with this tumor. Morphologically this tumor may mimic metastatic breast carcinomas or benign histiocytic lesions. Therefore, it would be challenging to distinguish them since a timely accurate diagnosis may have a great impact on patient management. Considering the poor prognosis of these lesions, their diagnosis and differentiation should be prioritized. 
